# Transcriptomic profiling reveals a pronociceptive role for angiotensin II in inflammatory bowel disease

**DOI:** 10.1097/j.pain.0000000000003159

**Published:** 2024-01-29

**Authors:** James P. Higham, Charity N. Bhebhe, Rohit A. Gupta, Michael M. Tranter, Farah M. Barakat, Harween Dogra, Natalie Bab, Eva Wozniak, Katie H. Barker, Catherine H. Wilson, Charles A. Mein, Tim Raine, James J. Cox, John N. Wood, Nicholas M. Croft, Paul D. Wright, David C. Bulmer

**Affiliations:** a Department of Pharmacology, University of Cambridge, Cambridge, United Kingdom; b Blizard Institute, Barts and the London School of Medicine and Dentistry, Queen Mary University of London, London, United Kingdom; c Genome Centre, Barts and the London School of Medicine and Dentistry, Queen Mary University of London, London, United Kingdom; d Department of Gastroenterology, Addenbrookes Hospital, Cambridge University Teaching Hospitals, Cambridge, United Kingdom; e Wolfson Institute for Biomedical Research, University College London, London, United Kingdom; f LifeArc, SBC Open Innovation Campus, Stevenage, United Kingdom

**Keywords:** Visceral pain, Inflammatory bowel disease, Colitis, Angiotensin II, AT_1_ receptors, Nociception, Angiotensin receptor blocker

## Abstract

Visceral pain is a leading cause of morbidity in inflammatory bowel disease (IBD), contributing significantly to reduced quality of life. Currently available analgesics often lack efficacy or have intolerable side effects, driving the need for a more complete understanding of the mechanisms causing pain. Whole transcriptome gene expression analysis was performed by bulk RNA sequencing of colonic biopsies from patients with ulcerative colitis (UC) and Crohn's disease (CD) reporting abdominal pain and compared with noninflamed control biopsies. Potential pronociceptive mediators were identified based on gene upregulation in IBD biopsy tissue and cognate receptor expression in murine colonic sensory neurons. Pronociceptive activity of identified mediators was assessed in assays of sensory neuron and colonic afferent activity. RNA sequencing analysis highlighted a 7.6-fold increase in the expression of angiotensinogen transcripts, *Agt*, which encode the precursor to angiotensin II (Ang II), in samples from UC patients (*P* = 3.2 × 10^−8^). Consistent with the marked expression of the angiotensin AT_1_ receptor in colonic sensory neurons, Ang II elicited an increase in intracellular Ca^2+^ in capsaicin-sensitive, voltage-gated sodium channel subtype Na_V_1.8-positive sensory neurons. Ang II also evoked action potential discharge in high-threshold colonic nociceptors. These effects were inhibited by the AT_1_ receptor antagonist valsartan. Findings from our study identify AT_1_ receptor-mediated colonic nociceptor activation as a novel pathway of visceral nociception in patients with UC. This work highlights the potential utility of angiotensin receptor blockers, such as valsartan, as treatments for pain in IBD.

## Abstract 2

Supplemental Digital Content is Available in the Text.

Using transcriptomic profiling combined with physiological and genetic dissection of nociceptor signalling, we identify angiotensin II as a putative mediator of nociception in colitis.

## 1. Introduction

Despite the marked progress in our understanding of the pathophysiology of inflammatory bowel disease (IBD), including ulcerative colitis (UC) and Crohn's disease (CD), abdominal pain continues to make a significant contribution to disease morbidity and lowered quality of life. As such, there is an unmet clinical need for the rational development of novel visceral analgesics to treat pain during colitis.

Abdominal pain during colitis develops due to the activation or sensitisation of nociceptors by mediators released from the inflamed bowel. The prolonged activation of gastrointestinal nociceptors leads to the development of visceral hypersensitivity which results in the perception of pain in response to innocuous stimuli, such as bowel movements, and the amplification of pain in response to noxious stimuli. A more detailed understanding of the mediators and mechanisms driving visceral nociception is imperative to facilitating the identification of novel drug targets for the treatment of abdominal pain.

In response to this challenge, we examined gene transcript expression in human colonic biopsies to generate a map of the cell types, signalling pathways, and pathophysiological processes underpinning UC and CD. In addition, we identified mediators with the potential to stimulate visceral nociceptors (ie, those for which receptors are expressed by murine colonic nociceptors). These data verified previous reports showing elevated expression of angiotensinogen (Agt) mRNA in the inflamed bowel,^10^ particularly in UC,^25^ which encodes the precursor to angiotensin II (Ang II). Given the expression of the angiotensin AT_1_ receptor in putative colonic nociceptors, we hypothesised that Ang II could be a mediator of visceral nociception during inflammation.

Although circulating Ang II protein levels are unchanged in IBD, a marked increase is found within the inflamed bowel,^10^ where Ang II concentrations correlate with endoscopically graded bowel inflammation.^19^ This local production of Ang II may be driven by upregulated cathepsin G,^25^ released by neutrophils,^6^ which cleaves Agt and Ang I to form Ang II,^27^ though renin and angiotensin converting enzyme (ACE) are also present in abundance in the intestine. Leukocytes synthesise and release Agt,^12^ providing a source of substrate for Agt-cleaving enzymes in inflamed tissue. Indeed, both stimulation of the renin–angiotensin system and Ang II infusion promote colitis in experimental models,^33^ while blockade of AT_1_ receptor signalling ameliorates colonic inflammation in both mice with experimental colitis^20,30,34^ and humans with IBD.^18,33^

Despite an increasingly clear role for Ang II and AT_1_ in colonic inflammation, little is known about their function in pain arising from the viscera. The expression of AT_1_ receptors in the *Mrgprd*^+^ population of nonpeptidergic (NP) nociceptors^15,38^ points to a role in visceral nociception consistent with a recent report showing that *Mrgprd*^+^ positive nociceptors are important in visceral nociceptive signalling in mouse.^2^ Consequently, the aim of this study was to determine the pronociceptive properties of Ang II and AT_1_.

## 2. Methods and materials

### 2.1. Colonic biopsies

Colonic biopsies were taken after legal guardian consent from paediatric patients undergoing colonoscopy as part of their routine medical care. For patient data, see supplemental data 1 (available at http://links.lww.com/PAIN/B984). Biopsies were taken from patients undergoing diagnostic colonoscopy at the Royal London Hospital. Colonic biopsies were taken from the sites of inflammation in UC patients (n = 9 biopsies from N = 9 patients), and CD patients divided into drug naïve (CDN) (n = 8 biopsies from N = 7 patients), and treatment refractory (CDT) (n = 11 biopsies from N = 7 patients) subgroups, with all groups reporting abdominal pain in the 4 weeks before endoscopy. Biopsies were also taken from the sigmoid colon of patients who reported symptoms of abdominal pain in the 4 weeks before endoscopy but showed no signs of inflammation on investigation and were subsequently diagnosed as having recurrent abdominal pain (RAP) (n = 21 biopsies from N = 16 patients). Finally, biopsies were also taken from the sigmoid colon of noninflamed control patients (n = 14 biopsies from N = 8 patients), who reported no abdominal pain in the 4 weeks before colonoscopy and showed no evidence of inflammation after endoscopy. A maximum of 2 biopsies (from different sites) were taken from each patient. It was not possible to obtain 2 biopsies from each patient, and as such, each biopsy was treated as an individual biological sample and underwent independent analysis. Details of the number of biopsies used and patients included for each clinical group are outlined in supplemental data 1 (available at http://links.lww.com/PAIN/B984). Ethical approval for the study was provided by the East London and The City Health Authority Research Ethics Committee (REC# P/01/023). Biopsy samples were collected in modified Krebs/HEPES buffer from which supernatants were taken for study in a separate series of experiments, following which samples were transferred to RNAlater and stored at −80°C until processing for RNA sequencing.

### 2.2. RNA sequencing of colonic biopsies

RNA was isolated from 30 mg of human colonic tissue using an RNeasy mini tissue kit (Qiagen, Hilden, Germany) with DNase treatment. The resulting concentration of RNA was determined by NanoDrop 1000 (Thermo, Waltham, MA). RNA integrity was assayed with the Bioanalyzer (Agilent, Santa Clara, CA). Only RNA of suitable quality (ie, RNA integrity number = 8; rRNA ratio [28S/18S] = 2) was used for RNA sequencing. Libraries were generated from 100 ng total RNA with NEBNext Ultra with polyA selection (NEB). RNA sequencing was performed at the Queen Mary University of London Genome Centre (https://www.qmul.ac.uk/blizard/genome-centre/) with Illumina NextSeq500 with an average of 44 million 75 bp paired end reads generated per sample. The quality of the sequencing reads (fastq files) was assessed by FastQC (version 0.11.2). The reads were trimmed for adaptor sequences and poor-quality reads with Trim Galore (version 0.3.7). Two trimming phases were applied, the first to remove adaptors and the second to remove poly G sequences. The quality of the trimmed sequences was reassessed with FastQC (version 0.11.2). After satisfactory quality control, trimmed sequences were aligned to the coding regions of the human reference genome (GRCh37) with TopHat2 (version 2.0.13) and bowtie2 (version 2.2.3). Transcript abundance was then calculated by HTSeq-counts software (version 0.6.0). Unadjusted transcript abundance was then exported to the R environment (version 3.1.2) for exploratory data analysis and differential expression analyses. The principal component analysis (PCA) and distance between samples from DESeq2 (version 1.6.3) were used to assess the dispersion and categorization of samples. Differential expression analysis was investigated with edgeR (version_3.8.6). Genes with low counts and expressed in only one sample per category were removed from further analysis. The calcNormFactors function was used to calculate the normalization factors to account for library sizes. Samples were then investigated for differences between disease states. Dispersion was calculated by using the functions estimateCommonDisp and estimateTagwiseDisp. The false discovery rate was used to correct for multiple comparisons between groups.

### 2.3. Enrichment analysis

**Figure 1.**
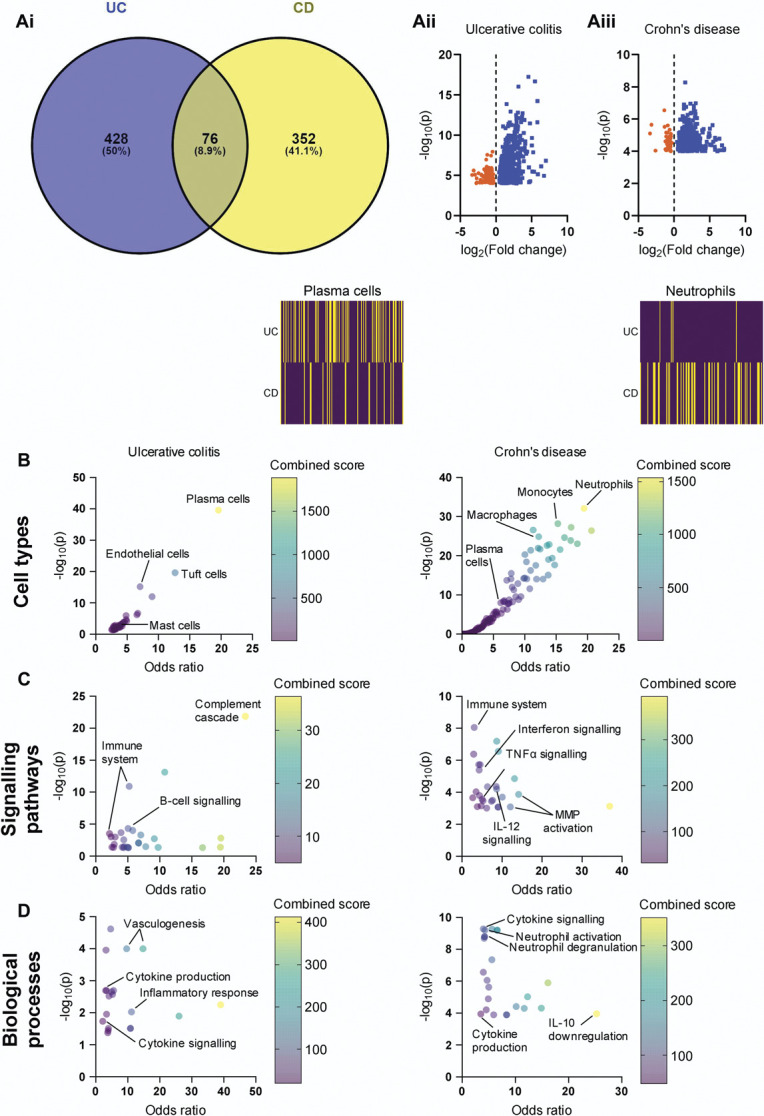
RNAseq and enrichment analysis of colonic biopsies from patients with UC or CD. (A) (*i*) Venn diagram showing the overlap between genes upregulated in UC (blue) and CD (yellow) compared with noninflamed controls. (ii and iii) Volcano plots showing all genes upregulated (blue) or downregulated (orange) in UC and CD (*P* ≤ 0.0001). (B) Bubble plots depicting the enrichment of gene ontology terms corresponding to different cell types in UC (left) or CD (right). Colours represent the combined score, providing a metric of the extent to which genes corresponding to a given ontology term (eg, cell type) are overrepresented (applies to (B–D)). *Inset above*: heatmaps showing the annotated gene sets used to identify plasma cells (l*eft*) or neutrophils (*right*); yellow indicates gene upregulation in biopsy tissue. In UC, 52/166 genes annotated to plasma cells were upregulated; in CD, 42/151 genes annotated to neutrophils were upregulated. (C) Bubble plots depicting the enrichment of gene ontology terms corresponding to different signalling pathways in UC (*left*) or CD (*right*). (D) Bubble plots depicting the enrichment of gene ontology terms corresponding to different biological processes in UC (*left*) or CD (*right*). CD, Crohn's disease; UC, ulcerative colitis.

**Figure 2.**
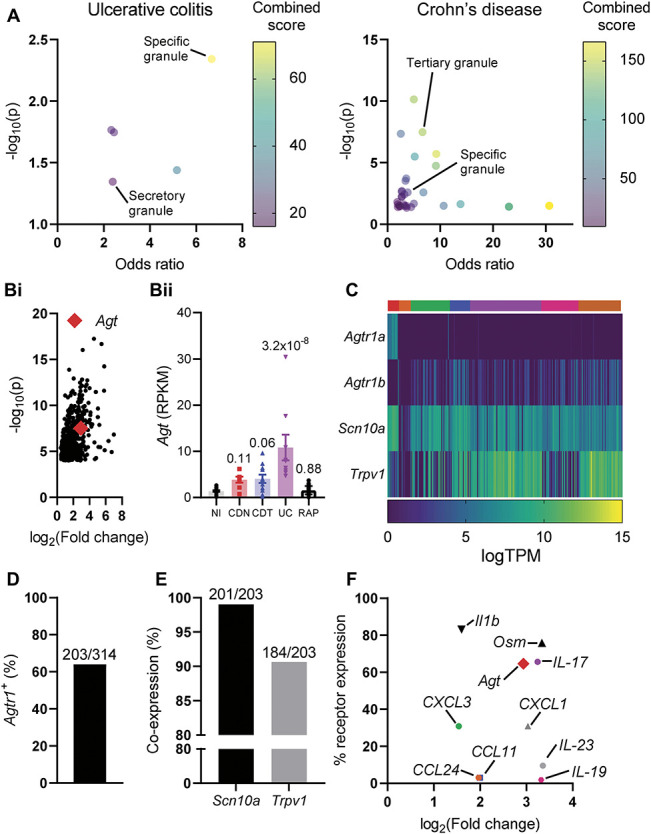
*Agt* mRNA was elevated in UC biopsies, and receptors for Ang II are expressed on colonic nociceptors. (A) Bubble plots depicting the enrichment of gene ontology terms corresponding to different subcellular compartments in UC (*left*) or CD (*right*). (B) (i) Scatter plot showing the average fold change (relative to noninflamed control) for all genes enriched in UC biopsies. *Agt* is highlighted (red diamond). (ii) *Agt* RPKM for each biopsy sample across all patient groups. Benjamini–Hochberg corrected *P* values displayed above bars. (C) Heatmap of gene expression for given genes in mouse colonic sensory neurons. TPM, transcripts per million (expressed as log[TPM]). Data in (C–F) redrawn from Hockley et al.^15^ Colours above the heatmap represent the assigned neuronal population from Ref. 15. Red, mixed thoracolumbar and lumbar splanchnic (“mixed”) nonpeptidergic afferents; orange and green, mixed neurofilament-expressing afferents; blue and purple, mixed peptidergic afferents; pink, lumbar splanchnic neurofilament-expressing afferents; brown, lumbar splanchnic peptidergic afferents. (D) The proportion of mouse colonic sensory neurons expressing either *Agtr1a* or *Agtr1b* transcripts. (E) The proportion of *Agtr1*-expressing colonic sensory neurons coexpressing the nociceptive neuronal markers *Scn10a* or *Trpv1* in mouse. (F) Scatter plot showing the fold change (relative to noninflamed controls) for a panel of mediators elevated in UC colonic biopsies against the proportion of mouse colonic sensory neurons which express a putative receptor for each mediator. CD, Crohn's disease; RPKM, reads per kilobase per million; UC, ulcerative colitis.

Enrichment analysis was performed to identify signals of enriched cell types (Panglao DB Augmented 2021), signalling pathways (Bio Planet 2019), biological processes (GO Biological Processes 2021), and subcellular compartments (GO Cellular Compartments 2021) within the sets of genes upregulated in UC and CD (cutoff for upregulation, *P* < 0.0001) using EnrichR (https://maayanlab.cloud/Enrichr/). In Figures 1 and 2, the odds ratio is the abundance of genes corresponding to a particular enrichment term in the upregulated gene set compared with background. Significance of enrichment of particular terms was determined using the Fisher exact test with Benjamini–Hochberg post-tests (terms were denoted as significantly enriched if *P* < 0.05). The combined score for an enrichment term is the product of the natural logarithm of the Benjamini–Hochberg adjusted *P* value and the z-score for the deviation from the expected rank of the term, providing further stratification of enriched terms.

### 2.4. Animals

All animal work was performed in accordance with the Animals (Scientific Procedures) Act 1986 with prior approval under Home Office License PPL 70/7382. Mice were housed in cages of up to 6 littermates under a 12-hour light/dark cycle with enrichment (eg, igloos and tunnels) and ad libitum access to food and water. Unless stated otherwise (Fig. 5J), all mice used were male aged 8 to 14 weeks on a C57Bl/6 background. Na_V_1.8^Cre^ (Jackson Laboratories stock 036564),^26^ ROSA26^CAG-flox-stop-tdTom^ (Jackson Laboratories stock 007905),^24^ and ROSA26^flox-stop-eGFP-DTA^ (Jackson Laboratories stock 032087)^1,17^ mouse lines and genotyping protocols have been described previously. Tmem45b^Cre^ and Advillin^flox-stop-tdTom-DTA^ mouse lines were generated and characterised recently.^40^

### 2.5. Culture of sensory neurons from dorsal root ganglia

Murine dorsal root ganglia (DRG, T12-L6) were harvested and incubated with Lebovitz L-15 Glutamax media (Invitrogen, Waltham, MA) containing 1 mg/mL type 1A collagenase (Sigma Aldrich, St Louis, MO) and 6 mg/mL bovine serum albumin (BSA, Sigma-Aldrich) for 15 minutes (37°C, 5% CO_2_). Dorsal root ganglia were subsequently incubated with L-15 media containing 1 mg/mL trypsin (Sigma-Aldrich) and 6 mg/mL BSA for 30 minutes (37°C, 5% CO_2_). Dorsal root ganglia were gently triturated using a P1000 pipette tip and pelleted by centrifugation at 100*g* for 30 seconds. The supernatant (containing dissociated cells) was collected, and trituration was repeated 5 times. The collected supernatant was centrifuged at 100*g* for 5 minutes, and pelleted cells were resuspended in L‐15 media supplemented with 10% (vol/vol) foetal bovine serum, 2.6% (vol/vol) NaHCO_3_, 1.5% (vol/vol) D-glucose, and penicillin/streptomycin and plated on to laminin-coated and poly-D-lysine–coated coverslips (MatTek, Ashland, MA). Cells were incubated at 37°C in 5% CO_2_ and were used for imaging after no more than 24 hours.

### 2.6. Ca^2+^ imaging of cultured sensory neurons

Cells were loaded with 10 µM Fluo-4-AM diluted in bath solution (in mM: 140 NaCl, 4 KCl, 1 MgCl_2_, 2 CaCl_2_, 4 D-glucose, and 10 HEPES; pH 7.35-7.45) by incubation for 30 to 45 minutes (room temperature, shielded from light). After incubation, coverslips were washed with bath solution and mounted on the stage of an inverted microscope (Nikon Eclipse TE2000S). For studies using antagonists, cells were preincubated with drug-containing solution (200 µL) for 10 minutes before imaging. During imaging, cells were superfused with bath solution at ∼0.5 mL/minute using a gravity-fed perfusion tip.

Images were captured using a CCD camera (Retiga Electro, Photometrics, Tucson, AZ) at 2.5 Hz with 100 ms exposure. Fluo-4 was excited by a 470 nm light source (Cairn Research, Kent, UK), and emission at 520 nm was recorded using µManager. Where multiple drugs were added to the same coverslip, at least 3 minutes elapsed between applications. At the end of each experiment, 50 mM KCl was applied to identify viable neurons and enable the normalisation of fluorescence.

Image analysis was performed using ImageJ. Regions of interest were manually drawn around cells, and average pixel intensity per neuron per frame was measured and analysed using custom-written scripts in RStudio. After the subtraction of background fluorescence, values (F) were normalised to baseline fluorescence (10 seconds before drug application) and the maximal fluorescence during KCl application (F_pos_) such that 0 F/F_pos_ and 1 F/F_pos_ represent baseline and maximal fluorescence in KCl, respectively. Only cells which exhibited a stable baseline and a rise in fluorescence of >5% over baseline during KCl application were included in analysis. No difference in the magnitude of the response to KCl was observed between experimental groups. Neurons were classed as responsive to a particular drug if fluorescence >0.1 F/F_pos_ was attained.

### 2.7. Magnetic activated cell sorting of cultured sensory neurons

Dorsal root ganglia from 2 to 3 mice were isolated and cultured as above, but trypsin incubation was omitted, and DRG were incubated with collagenase (1 mg/mL with 6 mg/mL BSA) for 45 minutes. Pelleted neurons were washed in 2 mL Dulbecco phosphate‐buffered saline (DPBS, containing 0.9 mM CaCl_2_ and 0.5 mM MgCl_2_) and centrifuged for 7 minutes (100*g*). Pelleted cells were resuspended in magnetic activated cell sorting (MACS) rinsing solution (120 μL, Miltenyi Biotec, Cologne, Germany), supplemented with 0.5% w/v BSA (sterile filtered at 0.2 μM), and incubated (5 minutes at 4°C) with a biotin‐conjugated non‐neuronal antibody cocktail (30 μL, Miltenyi Biotec). Dulbecco phosphate‐buffered saline was added up to a volume of 2 mL, and the suspension was centrifuged for 7 minutes at 100*g*. The pellet was resuspended in 120 μL MACS rinsing solution with 30 μL biotin‐binding magnetic beads (Miltenyi Biotec) and incubated for a further 10 minutes at 4°C, before being topped up to 500 μL with MACS rinsing buffer.

The cell suspension was filtered by gravity through a magnetic column (LD column, Miltenyi Biotec) primed with 2.5 mL MACS rinsing solution. After the addition of the cell suspension, 1 mL MACS rinsing solution was used to collect the remnants of the cell suspension and passed through the column before a final wash. The 5 mL eluted was centrifuged for 7 minutes at 100*g*, and the final pellet was resuspended in supplemented L‐15 medium, before plating on 35-mm poly‐D‐lysine–coated glass bottom culture dishes further coated with Matrigel (diluted 1:10 in L‐15 medium). Cells were incubated in supplemented L‐15 media at 37°C in 5% CO_2_ and were used for imaging after 48 hours (L-15 media was replaced after 24 hours).

### 2.8. Immunocytochemistry of cultured sensory neurons

Dorsal root ganglia neurons were cultured as above and seeded onto 12-mm coverslips coated in poly-D-lysine and laminin. After 24 to 48 hours in culture, cells were fixed at room temperature in 4% paraformaldehyde (10 minutes) and washed in PBS. Cells were permeabilized with 0.05% Triton‐X100 for 5 minutes at room temperature. Cells were washed again in PBS, and blocking buffer (1% goat serum in 0.2% Triton‐X100) was applied for 30 minutes. Cells were incubated with a rabbit anti–βIII‐tubulin primary antibody (1:1000, Abcam, Cambridge, UK: ab18207; RRID: AB_444319) for 3 hours at room temperature.

After primary antibody incubation, cells were washed in PBS and incubated with an Alexa Fluor‐568 goat anti‐rabbit secondary antibody diluted in PBS (1:1000, Invitrogen: A11008; RRID: AB_143165) plus 4′‐6‐diamidino‐2‐phenylindole (DAPI; 1:1000, Abcam) for 1 hour at room temperature. After a final wash, coverslips were mounted, cell side down, on 25 × 75 × 1-mm glass slides using Mowiol 4-88 mounting medium (Sigma‐Aldrich: 81381). Mounting medium was set at 4°C, and slides were imaged within 2 hours.

Slides were imaged using an Olympus BX51 microscope. Fluorophores were excited with 568 nm (Alexa Fluor‐568) or 350 nm (DAPI) light sources. Images were captured on a Qicam CCD camera (QImaging) with either 100 ms (Alexa Fluor‐568) or 50 ms (DAPI) exposure and false coloured (βIII‐tubulin, green; DAPI, blue). No βIII‐tubulin staining was observed when the primary antibody was omitted (data not shown).

Images were analysed using ImageJ, as previously described.^14^ An automatic “minimum error” threshold was applied to 8‐bit images of βIII‐tubulin or DAPI staining to distinguish background from objects of interest. Binary and raw images were manually compared, and the threshold manually adjusted to ensure all regions of interest were captured. The threshold was invariably placed within the first minimum after the major peak of the image histogram. Binary images then underwent watershed segmentation to separate distinct objects in close apposition. Identified objects, positive for βIII‐tubulin and/or DAPI, were automatically counted using ImageJ, and a ratio of βIII‐tubulin–positive cells (neurons) to DAPI‐positive cells (neurons and non-neuronal satellite cells) was calculated.

### 2.9. Electrophysiological recording from the mouse lumbar splanchnic nerve

For both multiunit and single-unit recording, the colorectum (from splenic flexure to anus) with the associated lumbar splanchnic nerve was isolated and removed. The colorectum was flushed and transferred to a tissue bath before being cannulated and both luminally perfused (200 µL/minute) and serosally superfused (7 mL/minute; 32-34°C) with Krebs buffer (in mM: 124 NaCl, 4.8 KCl, 1.3 NaH_2_PO_4_, 25 NaHCO_3_, 1.2 MgSO_4_, 11.1 D-glucose, and 2.5 CaCl_2_) supplemented with atropine (10 µM) and nifedipine (10 µM) to block smooth muscle activity. Luminal pressure was maintained between 2 to 5 mm Hg (Neurolog NL108, Digitimer Ltd, Welwyn Garden City, Hertfordshire, UK). Activity from isolated bundles (or teased fibres) of the lumbar splanchnic nerve (rostral to the inferior mesenteric ganglion) was recorded using borosilicate glass suction electrodes. Signals were amplified (gain, 5 kHz), band pass filtered (100-1500 Hz, Neurolog, Digitimer Ltd), and digitally filtered for 50 Hz noise (Humbug, Quest Scientific, Vancouver, Canada). Signals were digitised (20 kHz, Micro1401, Cambridge Electronic Design, Cambridge, UK) and recorded using Spike2 (Cambridge Electronic Design). Nerve discharge was quantified by determining the number of field potentials which were greater in magnitude than twice the background noise (typically 60-80 µV). Changes in nerve discharge were calculated by subtracting baseline firing (average of 5 minutes before drug application) from activity during drug application, with the peak change being found within the 10 minutes after drug application. In teased fibre experiments, single units were identified by waveform matching, allowing the properties of individual fibres to be determined.^16^

### 2.10. Statistics

All data were scrutinised to verify that they met the assumptions of parametric analyses. Normality was assessed using the Shapiro–Wilk test and homogeneity of variances with F-tests; heterogeneity of variances was corrected using Welch's correction where appropriate. Where the assumptions required for parametric analyses were not met, rank-based, nonparametric alternatives were used. Sample sizes were not prespecified before data acquisition, but intergroup comparisons were decided before data were obtained, and all statistical tests performed are reported. Data are presented as mean ± standard error (SEM). *P* values are stated in figures, or cutoffs are denoted as *P < 0.05, **P < 0.01, ***P < 0.001.

## 3. Results

### 3.1. RNAseq analysis of colonic biopsies provides insight into the mechanisms underpinning inflammatory bowel disease

The gene expression profile of colonic biopsies taken from paediatric patients (Supplemental Data 1, available at http://links.lww.com/PAIN/B984) diagnosed with either UC or CD (treatment naïve) were compared with those from noninflamed controls to identify differentially expressed genes in the inflamed bowel (cutoff, *P* ≤ 0.0001, Supplemental Data 2 and 3, available at http://links.lww.com/PAIN/B993, http://links.lww.com/PAIN/B994, http://links.lww.com/PAIN/B995). In UC biopsies, 504 genes were upregulated, while 428 genes were upregulated in CD (Fig. 1Ai). 76 upregulated genes were shared between UC and CD. The treatment-naïve CD (CDN) cohort was also compared with a treatment refractory CD (CDT) group with inflammation on endoscopy. In colonic biopsies from the CDT group, 329 genes were upregulated compared with noninflamed controls; only 25 were shared with those upregulated in CDN patients (Supplemental Figure 1A and B, available at http://links.lww.com/PAIN/B984; Supplemental Data 3, http://links.lww.com/PAIN/B995). We also examined genes downregulated compared with noninflamed biopsies (Fig. 1Aii–iii; Supplemental Data 3, http://links.lww.com/PAIN/B995). In CDN, 51 genes were downregulated, compared with 164 in UC, 31 of which were shared between conditions. 106 genes were downregulated in the CDT cohort, and 20 of these were in common with CDN patients. Biopsies taken from patients with recurrent abdominal pain showed no gene upregulation relative to noninflamed controls, and only 6 genes were significantly downregulated (Supplemental Data 3, http://links.lww.com/PAIN/B995).

The upregulated gene sets in UC, CDN, and CDT biopsies were compared with annotated gene sets of known biological function (gene ontologies), and enrichment analysis was performed to infer the processes driven by the upregulated genes^5^ (Supplemental Data 4, http://links.lww.com/PAIN/B996). There was a striking difference in the immune cell types enriched in UC and CDN (Fig. 1B). The genes upregulated in UC indicated the elevated presence of plasma cells (*P* = 2.8 × 10^−40^), tuft cells (*P* = 2.5 × 10^−20^), and mast cells (*P* = 0.0025), while neutrophils (*P* = 8.2 × 10^–33^) and macrophages (*P* = 1.4 × 10^–25^) were elevated in CDN (Fig. 1B). Given the marked difference in upregulated genes between CDN and CDT biopsies, it is unsurprising that there was also a distinct difference in the cell types present in these groups. There was a reduced enrichment of genes indicating the presence of macrophages and neutrophils in CDT biopsies compared with CDN biopsies (Supplemental Figure 1C–E, available at http://links.lww.com/PAIN/B984). However, there was an enrichment of various subsets of T cells in CDT biopsies, such as T_memory_ cells (*P* = 7.6 × 10^−25^) and T_regulatory_ cells (*P* = 2.0 × 10^−19^; Supplemental Figure 1C–E, available at http://links.lww.com/PAIN/B984).

The differences in the cell types present are partially reflected in the differential enrichment of signalling pathways (Fig. 1C) and biological processes (Fig. 1D) in UC and CDN. For example, signatures of B-cell signalling are present in UC (*P* = 4.9 × 10^–5^), while signatures of tumour necrosis factor α (TNFα, *P* = 2.7 × 10^–4^) and matrix metalloprotease (MMP, *P* = 1.4 × 10^–4^) signalling are elevated in CDN (Fig. 1C). In agreement with this, multiple members of the MMP family are upregulated in CDN (MMP2, 4.1-fold; MMP8, 122.6-fold; MMP9, 6.4-fold; MMP14, 2.1-fold; MMP25, 7.6-fold). Neutrophils are a major source of both TNFα and MMPs, and consistently, genes associated with neutrophil activation (*P* = 6.4 × 10^–10^) and degranulation (*P* = 1.6 × 10^–9^) are enriched in CDN (Fig. 1D). Signatures of interferon signalling were also identified in CDN biopsies (*P* = 4.1 × 10^–6^, Fig. 1C).

Enrichment analysis permitted insight into the subcellular compartments involved in signalling events driving UC and CDN. In both diseases, genes associated with secretory granules were enriched (Fig. 2A). We therefore sought to identify secreted mediators for which there are receptors expressed on colonic nociceptors. In our biopsy data, *Agt* mRNA was elevated 7.6-fold in UC (RPKM_noninflamed_ = 1.42 ± 0.17, RPKM_UC_ = 10.83 ± 2.8, *P* = 3.2 × 10^−8^) relative to noninflamed controls (Fig. 2B). While the source of *Agt* remains unclear, markers of plasma cells and tuft cells—both highly enriched in UC—correlate with the levels of *Agt* across all patient groups (Supplemental Figure 2, available at http://links.lww.com/PAIN/B984).

Receptors for Ang II, the major biologically active metabolite of Agt, are expressed by a large subpopulation of murine colonic sensory neurons (Fig. 2C). In total, 203 of 314 colonic sensory neurons expressed *Agtr1a* and/or *Agtr1b*, the genes encoding the 2 isoforms of AT_1_ in mouse (Fig. 2D). *Agtr2*, encoding AT_2_, is not expressed by sensory neurons.^15,38^ Of 203 neurons expressing a receptor for Ang II, 201 (99.0%) and 184 (80.8%) coexpressed the nociceptive neuronal markers *Scn10a* and *Trpv1*, respectively (Fig. 2E).

To compare Ang II to other potential targets for investigation, we considered a panel of secreted mediators elevated in UC and examined their fold change and the proportion of colonic sensory neurons expressing a putative receptor for the mediator (Fig. 2F). *Agt* was compared with *Il1b*, *Osm*, *CXCL1*, *CXCL3*, *CCL11*, *CCL24*, *IL17*, *IL19*, and *IL23*. Receptors for CCL11, CCL24, IL-19, and IL-23 are expressed on only a small subset (<10%) of colonic afferents and were not chosen for further investigation. Receptors for IL-17, oncostatin M, and IL1β were expressed by 65.6%, 75.8%, and 83.1% of colonic afferents, respectively (Fig. 2F). It is known that these mediators interact with sensory neurons,^3,29,31,37^ while the effects of Ang II on sensory neurons are less clear, and as such, we have investigated whether Ang II exerts a stimulatory effect on nociceptive sensory neurons.

### 3.2. Ang II stimulates nociceptive neurons in vitro

**Figure 3.**
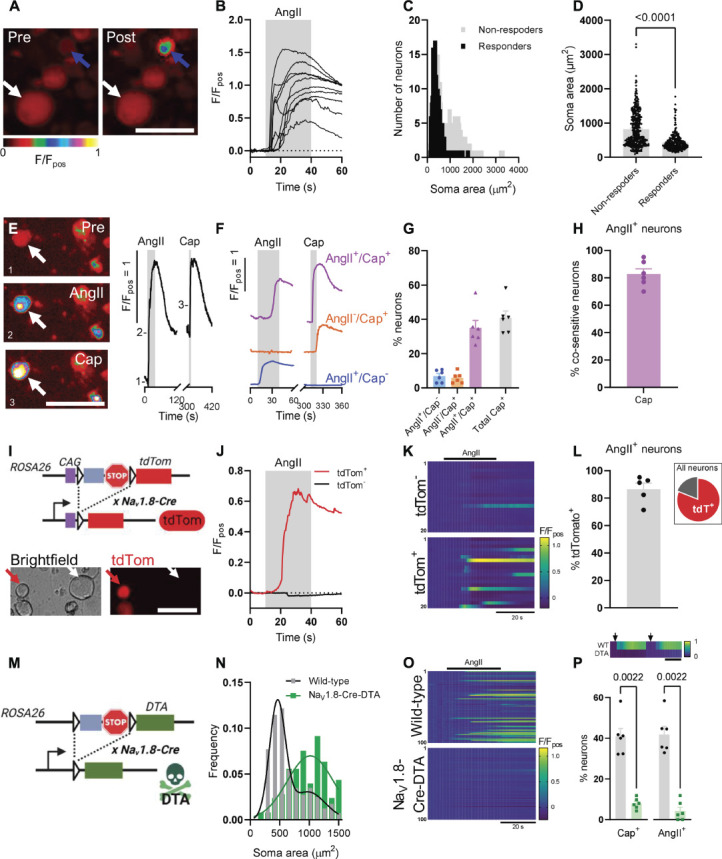
Properties of Ang II–sensitive sensory neurons in vitro. (A) False-coloured images depicting Fluo4 fluorescence before (left) and after (right) Ang II application. Blue and white arrows highlight exemplar Ang II–sensitive and Ang II–insensitive neurons, respectively. Scale bar: 50 µm. (B) Fluo4 fluorescence traces showing the response to Ang II application in 10 randomly selected Ang II–sensitive neurons. (C) Histogram of the neuronal soma area of Ang II–sensitive (black) and Ang II–insensitive (gray) neurons. (D) Grouped data showing the neuronal soma area for Ang II–sensitive and Ang II–insensitive neurons. N = 590 neurons (235 Ang II–sensitive and 355 Ang II–insensitive) derived from 4 independent cultures. Data analysed using a Mann–Whitney *U* test. (E) (*Left*) False-coloured images depicting Fluo4 fluorescence at baseline (top), during Ang II application (middle), and during capsaicin application (bottom). Scale bar: 50 µm. (*Right*) Fluo4 fluorescence trace showing the response to sequentially applied Ang II (30 seconds) and capsaicin (10 seconds) for the neuron highlighted by the white arrow (*left*). Numbers on the trace show where the corresponding images on the left were taken. (F) Exemplar Fluo4 fluorescence traces from neurons within each of the populations identified by sensitivity to Ang II and/or capsaicin. (G) Grouped data showing the respective proportional size of each of the identified populations. N = 221 neurons imaged across 6 coverslips from 4 independent cultures. (H) Cosensitivity to capsaicin of Ang II–sensitive neurons, derived from the data in G. (I) (*Top*) Generation of mice expressing tdTomato in Na_V_1.8-positive sensory neurons. A CAG promotor is placed upstream of the floxed (triangles) stop sequence and tdTom to ensure robust expression. A transcriptional stop site halts transcription upstream of tdTom in the absence of Cre. Excision of the floxed stop site by Na_V_1.8-Cre results in the labelling of Na_V_1.8-expressing neurons with tdTomato. Subsequent data (J–L) derived from 384 neurons from 3 independent cultures. (*Bottom*) Images showing exemplar tdTomato^+^ and tdTomato^−^ neurons. Scale bar: 30 µm. (J) Fluo4 fluorescence traces showing the response to Ang II for the neurons highlighted by red (tdTomato^+^) and white (tdTomato^−^) arrows in I (*bottom*). (K) Heatmaps showing Fluo4 fluorescence during the application of Ang II for 20 randomly selected tdTomato^−^ (*top*) and tdTomato^+^ (*bottom*) neurons. (L) Proportion of Ang II–sensitive neurons which expressed tdTomato and, hence, Na_V_1.8. (*Inset*) The proportion of all neurons imaged which expressed tdTomato (red). 384 neurons imaged across 5 coverslips from 3 independent cultures. (M) Generation of mice expressing DTA in Na_V_1.8-positive sensory neurons. Excision of the floxed stop site with Na_V_1.8-Cre results in the ablation of Na_V_1.8-expressing neurons. (N) Histogram of neuronal soma area for neurons derived from wild-type (black) and Na_V_1.8-Cre-DTA–expressing (green) mice. (O) Heatmaps showing Fluo4 fluorescence during the application of Ang II for 100 randomly selected wild-type (*top*) and Na_V_1.8-Cre-DTA (*bottom*) neurons. (P) Grouped data showing the proportion of neurons responding to capsaicin or Ang II in sensory neuron cultures derived from wild-type (black) or Na_V_1.8-Cre-DTA (green) mice. Wild-type: 312 neurons imaged across 6 coverslips from 4 independent cultures; Na_V_1.8-Cre-DTA: 183 neurons imaged across 6 coverslips from 2 independent cultures. Data analysed using two-tailed Mann–Whitney *U* tests. (*Inset, above*) Exemplar heatmaps showing the response of neurons from wild-type (WT) and Na_V_1.8-Cre-DTA (DTA) cultures to the sequential application of Ang II and capsaicin (arrows). Scale bar: 30 seconds. DTA, Diphtheria Toxin A Chain.

To ascertain whether Ang II may be involved in nociceptive signalling, sensory neurons from murine DRG were cultured and Ca^2+^ imaging was used to determine whether Ang II–sensitive neurons were putative nociceptors. A subpopulation of sensory neurons in vitro responded to Ang II (2 µM, 30 seconds application, Figs. 3A and B). Ang II evoked a rise in cytosolic Ca^2+^ in 39.8% of sensory neurons (590 neurons from 4 independent cultures), most of which were of a small soma area (Fig. 3C). Ang II–sensitive neurons had a soma area of 437 ± 16 µm^2^ compared with 821 ± 27 µm^2^ for Ang II–insensitive neurons (*P* < 0.0001, Fig. 3D). A reduced concentration of Ang II (100 nM) evoked a rise in cytosolic Ca^2+^ in a smaller proportion of sensory neurons (8.0 ± 2.2%, 363 neurons imaged across 4 coverslips from 4 independent cultures).

Sensitivity to capsaicin, an agonist of the nonselective cation channel TRPV1, is a hallmark of a subset of nociceptive neurons. Sequential application of Ang II and capsaicin (1 µM) was used to establish the cosensitivity between these compounds (Fig. 3E). Three responsive populations were identified: those which responded to both Ang II and capsaicin, those which responded to Ang II alone, and those which responded to capsaicin alone (Figs. 3F and G). Within the Ang II–sensitive population, 82.8 ± 3.8% of neurons were cosensitive to capsaicin (Fig. 3H), and Ang II–sensitive neurons accounted for 84.9 ± 3.4% of the capsaicin-sensitive population. The response to capsaicin was not sensitised nor the proportion of capsaicin-sensitive neurons increased, by preincubation with Ang II (data not shown), indicating that cosensitivity was not artificially elevated by the experimental protocol.

The voltage-gated Na^+^ channel, Na_V_1.8, is a key marker of nociceptive sensory neurons. To test whether Ang II–sensitive neurons expressed Na_V_1.8, we first labelled these neurons using Cre-dependent expression of tdTomato (Fig. 3I) and investigated the colocalisation of Ang II–evoked Ca^2+^ signals with tdTomato (Fig. 3J). Ang II preferentially stimulated Na_V_1.8-positive neurons (Fig. 3K): 86.6 ± 4.4% of Ang II–sensitive neurons expressed tdTomato (Fig. 3L). Of 384 neurons (from 3 independent cultures) imaged, 311 (81.0%) were tdTomato^+^ (soma area: 483 ± 15 µm^2^) and 73 were tdTomato^−^ (soma area: 832 ± 69 µm^2^) (Fig. 3L inset), in agreement with previous observations.^23,36,38^

To further ratify the stimulation of Na_V_1.8-expressing neurons by Ang II, these neurons were ablated by Cre-dependent expression of the Diphtheria Toxin A Chain (DTA, Fig. 3M). The expression of DTA in Na_V_1.8-positive neurons led to a paucity of small-sized sensory neurons in vitro (Fig. 3N) and a marked reduction in the response to Ang II (Fig. 3O). In wild-type cultures, 41.9 ± 3.9% of neurons responded to Ang II, whereas only 4.2 ± 1.9% responded in cultures from Na_V_1.8-Cre-DTA mice (*P* = 0.0022, Fig. 3P). The response to capsaicin was similarly attenuated (41.0 ± 3.9% vs 7.9 ± 1.1%, *P* = 0.0022, Fig. 3P), in line with a previous report.^1^ These data provide functional evidence that Ang II–sensitive sensory neurons in vitro exhibit key features of nociceptors.

### 3.3. AT_1_ is required for the neuronal response to Ang II

**Figure 4.**
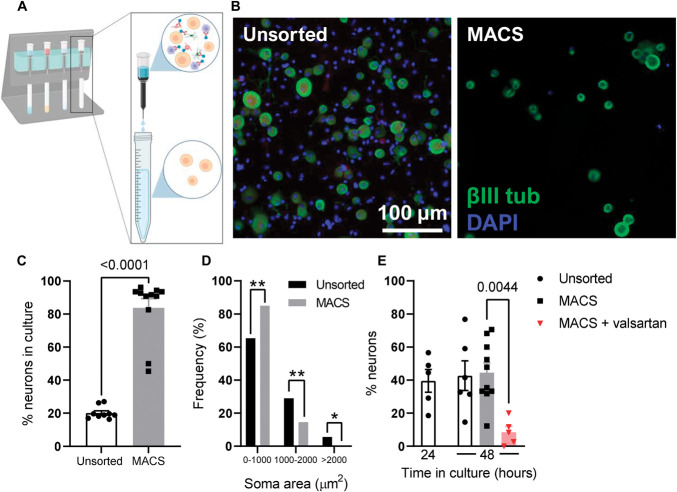
AT_1_R mediated the neuronal response to Ang II in vitro. (A) Schematic depicting MACS. Filter columns are loaded onto a magnetic stand (*left*) and the suspension of DRG-derived cells (with non-neuronal cells bound with magnetic beads) is passed through. Non-neuronal cells are retained in the column, and neuronal soma are eluted (*right*). (B) Images showing staining for βIII-tubulin (green) and DAPI (blue) in unsorted (*left*) and MACS (*right*) neuronal cultures. A loss of non-neuronal (DAPI^+^/βIII-tubulin^-^) cells is observed. (C) Grouped data showing the proportion of neurons in unsorted and MACS cultures. Unsorted: 9 coverslips from 3 independent cultures; MACS: 11 coverslips from 4 independent cultures. Data analysed using a 2-tailed Mann–Whitney *U* test. (D) Absolute frequency of neurons of given soma areas in unsorted and MACS cultures. Sorting of neurons led to an enrichment of smaller-sized neurons at the expense of neurons >1000 µm^2^. Unsorted: 298 neurons; MACS: 239 neurons. Data analysed using chi-square tests. (E) Grouped data showing the proportion of neurons responsive to Ang II application. Unsorted (24 hours): 5 coverslips from 4 independent cultures; unsorted (48 hours): 6 coverslips from 6 independent cultures; MACS: 10 coverslips from 9 independent cultures; MACS + valsartan: 5 coverslips from 5 independent cultures. Data analysed using a one-way ANOVA with Bonferroni post-tests. ANOVA, analysis of variance; DRG, Dorsal root ganglia; MACS, magnetic activated cell sorting.

There is a clear neuronal response to Ang II in in vitro sensory neurons from DRG. Sensory neuron cultures derived from DRG cannot help to resolve whether Ang II directly interacts with neurons because DRG cultures contain myriad non-neuronal cells. We used MACS^35,36^ of DRG cultures to remove non-neuronal cells (Fig. 4A). Magnetic activated cell sorting removed non-neuronal cells identified by positive DAPI staining and negative βIII-tubulin staining (Fig. 4B). In unsorted (control) cultures, neurons accounted for 20.2 ± 1.3% of all cells present, compared with 83.8 ± 5.5% after MACS (*P* < 0.0001, Fig. 4C). As reported previously,^36^ MACS resulted in a loss of large-sized sensory neurons (Fig. 4D).

After MACS, sensory neurons required ∼48 (rather than ∼24) hours in culture to properly adhere to the coverslip. This did not have any effect on the response to Ang II in unsorted cultures (*P* > 0.99, Fig. 4E). In unsorted cultures (after 48 hours in vitro), 42.7 ± 9.0% of neurons exhibited a rise in cytosolic Ca^2+^ after Ang II application (Fig. 4E). After MACS, a similar proportion of neurons responded to Ang II (44.5 ± 5.9%, *P* > 0.99, Fig. 4E). Preincubation of magnetically sorted sensory neurons with valsartan (1 µM), an AT_1_ antagonist, abrogated the response to Ang II, with only 8.5 ± 3.5% of neurons responding under these conditions (*P* = 0.0044, Fig. 4E). In unsorted DRG cultures, the AT_2_ antagonist, PD123319 (1 µM), had no effect on the neuronal response to Ang II (6 coverslips from 5 independent cultures, *P* = 0.27), while EMD66684 (100 nM; structurally distinct AT_1_ antagonist) did attenuate the proportion of neurons responding to Ang II (9 coverslips from 5 independent cultures, *P* = 0.008). These experiments suggest that at least a component of the neuronal response to Ang II is mediated by a direct interaction dependent on AT_1_ and not AT_2_.

### 3.4. Ang II stimulates colonic afferent activity through AT_1_

**Figure 5.**
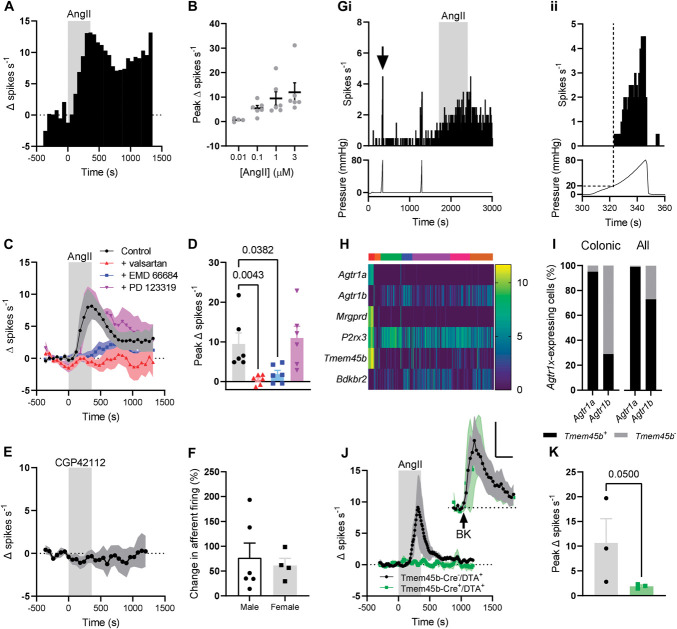
Ang II stimulated activity in the lumbar splanchnic nerve. (A) Exemplar rate histogram showing the change in afferent firing during the bath application of 1 µM Ang II (gray shaded area, 0-360 seconds). (B) Concentration–response relationship for the effect of Ang II on colonic afferent firing. 0.01 µM, N = 4; 0.1, 1 and 3 µM, N = 6. (C) Bath application of Ang II (gray shaded area, 0-360 seconds) evoked activity in the ex vivo LSN under control conditions (black trace). Preincubation of tissue with AT_1_ antagonist valsartan (red trace) or EMD66684 (blue trace) blunted the afferent response to Ang II application. PD123319, an AT_2_ antagonist, did not affect the afferent response to Ang II application (purple trace). Symbols show mean change in firing rate (Δ spikes s^−1^), and shaded areas show standard error. (D) Grouped data showing the peak change in afferent firing for the traces shown in C. Spike discharge induced by Ang II was attenuated by AT_1_, but not AT_2_, antagonists. Data from 6 independent recordings per condition. Data analysed using a Kruskal–Wallis test with Dunn post-tests. (E) Bath application of CGP42112 (gray shaded area, 0-360 seconds) did not evoke afferent firing. (F) Grouped data showing the peak percentage change in afferent firing (relative to baseline) evoked by Ang II in tissue from male (N = 6) and female (N = 4) mice. Data analysed using a two-tailed unpaired *t* test. (G) (i) Exemplar single-unit recording showing the response to ramp distension to 80 mm Hg and Ang II (gray shaded area). Rate histogram of single unit *(top)*; luminal pressure *(bottom)*. (ii) Expanded recording showing response to luminal distention marked by arrow in (i). Rate histogram of single unit *(top)*; luminal pressure *(bottom)*. Dotted lines show increased afferent firing at luminal pressures >20 mm Hg. (H) Heatmap of gene expression (log[TPM]) for given genes in mouse colonic sensory neurons, showing coexpression of *Agtr1a* and *Agtr1b* with the markers of nonpeptidergic sensory neurons, *Mrgprd*, *Tmem45b*, and *P2rx3*. Data redrawn from Ref. 15. Colours above the heatmap represent the assigned neuronal population from Ref. 15. Red, mixed thoracolumbar and lumbar splanchnic (“mixed”) nonpeptidergic afferents; orange and green, mixed neurofilament-expressing afferents; blue and purple, mixed peptidergic afferents; pink, lumbar splanchnic neurofilament-expressing afferents; brown, lumbar splanchnic peptidergic afferents. (I) *(Left)* Coexpression of *Agtr1a* or *Agtr1b* with the nonpeptidergic sensory neuronal marker *Tmem45b* in colonic afferent neurons. Data redrawn from Ref. 15. Coexpression of *Agtr1a* or *Agtr1b* with the nonpeptidergic sensory neuronal marker *Tmem45b* in all sensory neurons in the DRG *(right)*. Data redrawn from Ref. 38. Black: *Tmem45b*^+^; gray: *Tmem45b*^−^. (J) Bath application of Ang II (gray shaded area, 0-360 seconds) evoked activity in the ex vivo LSN from Tmem45b-Cre^-^/DTA^+^ mice (black trace), but the stimulatory effect of Ang II was reduced in the LSN from Tmem45b-Cre^+^/DTA^+^ mice (green trace). *Inset*: bath application of bradykinin (BK, 1 µM, arrow) evoked similar afferent activity in LSN from Tmem45b-Cre^-^/DTA^+^ and Tmem45b-Cre^+^/DTA^+^ mice. Scale: 500 seconds, 5 spikes s^−1^. Tmem45b-Cre^-^: 3 mice, 2 male, 1 female; Tmem45b-Cre^+^: 3 mice, 2 male, 1 female. Symbols show mean change in firing rate (Δ spikes s^−1^), and shaded areas show standard error. (K) Grouped data showing the peak change in afferent firing rate for the traces shown in (J). Data analysed with a one-tailed Mann–Whitney *U* test. DRG, Dorsal root ganglia; LSN, lumbar splanchnic nerve; TPM, transcripts per million.

We used whole-nerve suction electrode recording of the lumbar splanchnic nerve (LSN) innervating the distal colon to ascertain whether Ang II exerted a stimulatory effect on colonic afferents. Bath application of Ang II induced a concentration-dependent increase in afferent activity (Figs. 5A and B). One µM Ang II (N = 6) evoked a peak increase in afferent activity of 9.5 ± 2.8 spikes s^−1^ (Fig. 5C). To test the requirement of AT_1_ for the stimulatory effect of Ang II, tissue was preincubated with 1 of 2 structurally distinct AT_1_ antagonists, either valsartan (10 µM, N = 6) or EMD66684 (10 µM, N = 6). Valsartan reduced the peak increase in afferent activity to 0.48 ± 0.52 spikes s^−1^ (*P* = 0.0043, Figs. 5C and D). Similarly, EMD66684 attenuated the peak change in afferent activity to 1.9 ± 0.92 spikes s^−1^ (*P* = 0.038, Figs. 5C and D). Losartan (1 µM, N = 6), an inverse agonist of AT_1_, also attenuated the colonic afferent response to Ang II (*P* = 0.0032). PD123319 (10 µM, N = 6), an AT_2_-selective antagonist, did not affect peak afferent firing (*P* > 0.99, Figs. 5C and D) evoked by Ang II application. Consistently, an AT_2_-selective agonist, CGP42112 (1 µM, N = 6), failed to elicit any change in LSN activity (Fig. 5E). In female mice (N = 4), both baseline and Ang II–evoked LSN activity were lower compared with males (N = 6), although the proportional change in activity evoked by Ang II was similar between sexes (*P* = 0.70, Fig. 5F).

Isolating a small number of fibres from the LSN enabled the analysis of the properties of individual afferent fibres by waveform matching (Fig. 5G). Thirty-two individual afferent fibres (from 5 animals) were identified. Sixteen of the isolated afferent fibres responded to Ang II application, of which 11 were also responsive to colonic distension at pressures >20 mm Hg (Fig. 5G). In total, 20 of 32 isolated fibres responded to distension pressures >20 mm Hg.

Given the coexpression of *Agtr1a/b* with the markers of NP colonic afferents, such as *Mrgprd*, *P2rx3*, and *Tmem45b* (Fig. 5H^13^), we hypothesised that, in mice in which NP afferents had been ablated, the response to Ang II application would be attenuated. We used mice in which Tmem45b-Cre (Fig. 5I shows coexpression of *Agtr1a/b* with *Tmem45b*) was used to drive DTA expression, thereby ablating NP sensory neurons.^38,40^ Ang II application resulted in elevated spike discharge in the LSN from Tmem45b-Cre^-^ (control) animals (10.7 ± 4.9 spikes s^−1^, N = 3, Figs. 5J and K). However, the response to Ang II was attenuated in Tmem45b-Cre^+^ animals (Fig. 5J) with a peak change in afferent firing rate of 1.9 ± 0.24 spikes s^−1^ (*P* = 0.05, N = 3, Fig. 5K). The expression of the B_2_ bradykinin receptor lies mostly outside the NP colonic afferent population (Fig. 5H).^15^ Consistently, the peak change in afferent activity in response to bradykinin application was not affected by the ablation of NP neurons (*P* = 0.71, Fig. 5J *inset*).

## 4. Discussion

RNAseq analysis of colonic biopsies provided an insight into the cellular and molecular underpinnings of IBD, ratifying previous observations showing the differential enrichment of immune cell types between UC and CD. Ulcerative colitis biopsies were enriched with plasma cells and mast cells, both major effectors of the T_H_2-mediated immune response. However, CDN biopsies were enriched with macrophages indicative of a dominant T_H_1-mediated immune response, in line with signatures of interferon and TNFα signalling. Many genes were upregulated in CDT biopsies relative to noninflamed controls, indicative of the presence of multiple T-cell subpopulations in keeping with the treatment refractory nature of disease in this patient group. Furthermore, CDT and CDN biopsies differed substantially in their gene expression, suggesting that the bowel in treatment refractory CD patients represents a transcriptional state distinct from both treatment-naïve CD patients and noninflamed patients.

Comparison of differential gene expression for secreted mediators with cognate receptor expression in colonic neurons lead to the identification of Ang II/AT_1_–mediated activation of Na_V_1.8-positive colonic nociceptors as a putative pathway for visceral nociception in UC following elevated *Agt* expression.

This is consistent with work showing the correlation of Ang II with endoscopically graded inflammation in IBD^19^ and more recent reports of enhanced expression of *Agt* in colonic biopsies from patients with IBD.^10,25^ Our results align with those of multiple previous reports from paediatric patients in that we identified raised *Agt* expression in UC biopsies.^13,25,28^ One study comparing UC and CD colonic biopsies, like our study, found *Agt* was upregulated in only UC.^25^ However, an earlier report showed elevated levels of Ang II protein in both UC and CD biopsies.^19^ One may speculate that this disparity could be due to differences in the mechanisms underpinning Ang II production in CD and UC, but this is not yet clear. Many existing RNAseq studies of the inflamed bowel, as well as ours, centre on paediatric patient cohorts and, going forward, it will be interesting to see whether findings in paediatric patients are consistent with those in adult patients.

Furthermore, retrospective studies of IBD patients prescribed ACE inhibitors or angiotensin receptor (AT_1_) blockers (ARBs) revealed they experienced milder inflammation, reduced requirement for corticosteroid treatment, and a diminished risk of hospitalisation and surgical intervention^9,10,18^ in line with attenuated mucosal expression of inflammatory cytokines.^33^ Clinical findings are supported by data from animal models of colitis, which show elevated Ang II in the colonic mucosa after induction of experimental colitis,^20^ and a reduction in inflammation, diarrhoea, and mucosal proinflammatory cytokines after treatment with ACE inhibitors or ARBs, or in animals lacking AT_1_.^20,30,34^ In addition, stimulation of Ang II production by renin overexpression, or chronic Ang II administration, precipitated colitis in mice and increased mucosal expression of TNFα, IL-1β, IL-6, and IL-17.^33^

Despite the emerging proinflammatory role for Ang II in colitis, its role in visceral nociception has not been extensively studied. To address this, we first demonstrated that Ang II stimulates nociceptors using in vitro Ca^2+^ imaging. Ang II elevated cytosolic Ca^2+^ in small diameter DRG neurons cosensitive to the algogenic TRPV1 agonist capsaicin. Ang II–sensitive neurons also expressed Na_V_1.8, demonstrated by the colocalisation of Ang II–evoked Ca^2+^ transients with Na_V_1.8-tdTomato and the loss of Ang II–evoked Ca^2+^ transients after the ablation of Na_V_1.8-positive neurons. The direct activation of DRG neurons by Ang II was confirmed by repeating experiments after MACS to remove non-neuronal cells^35,36^ in which a comparable neuronal response with Ang II was observed. Given the loss of large-diameter neurons after MACS, one may expect an increase in the proportion of Ang II–sensitive neurons; this was not observed and may indicate a minor role for non-neuronal cells in the neuronal response to Ang II. What's more, this response (in the absence of non-neuronal cells) was abolished by pretreatment with valsartan, demonstrating that AT_1_ mediates the direct interaction between Ang II and nociceptors. Of note, this study did not recapitulate the findings of Shepherd et al.,^32^ who observed no increase in cytosolic Ca^2+^ in response to a similar concentration of Ang II in DRG sensory neurons, despite a more prolonged exposure time. The reasons underpinning this discrepancy are not immediately clear, although differences in neuronal culture conditions are apparent, most notably a possible reduction AT_1_ receptor expression^39^ over the longer culture time used by Shepherd and colleagues. In our study, unlike in Shepherd et al., sensory neurons were cultured in the absence of nerve growth factor (NGF). In central neurons, NGF blunted the effects of Ang II application,^8^ although it is not clear what effect NGF would have on the response of sensory neurons to Ang II. Nevertheless, our findings are consistent with functional studies in other cell types expressing AT_1_, including heterologous cell lines,^41^ adrenal chromaffin cells,^21^ and central neurons,^11^ which demonstrate AT_1_-mediated Ca^2+^ transients in response to Ang II.

TRPV1^+^ and Na_V_1.8^+^ neurons are known to mediate colonic hypersensitivity,^4,7^ indicating that their stimulation by Ang II may contribute to hypersensitivity and pain in the inflamed bowel. Consistent with this, we demonstrated that Ang II elicited a marked increase in colonic afferent activity in tissue from both male and female mice. In keeping with the expression of AT_1_, but not AT_2,_ receptors in colonic neurons,^15^ the Ang II–mediated increase in colonic afferent activity was attenuated by pretreatment with AT_1_, but not AT_2_, antagonists. The response to Ang II could not be recapitulated by administration of an AT_2_ agonist. Furthermore, teased fibre recordings confirmed that most colonic afferent fibres sensitive to Ang II were high threshold based on their cosensitivity to luminal distension at pressures >20 mm Hg. Although AT_1_ is known to be expressed on colon-innervating neurons, it is also expressed by other neurons (enteric neurons) and non-neuronal cells, such as enteroendocrine cells,^22^ within the gut. As such, it is highly likely that the contribution of Ang II to visceral nociception in IBD may be mediated through a variety of neuronal and non-neuronal pathways alongside the direct activation of sensory neurons revealed in this study. However, based on our preliminary data showing the loss of colonic afferent sensitivity to Ang II in tissue from mice in which the NP nociceptor population had been ablated, it would seem that the direct activation of AT_1_-expressing sensory neurons is a dominant pathway by which Ang II stimulates visceral nociceptors in mouse. Further work is needed to establish the conservation of this pronociceptive pathway in human and the relative contribution of this pathway in inflamed tissue.

In summary, we have used genetic profiling of inflamed IBD tissue and sensory neurons to identify a novel pathway for visceral nociceptor activation in the inflamed bowel, namely Ang II/AT_1_–mediated activation of Na_V_1.8-positive nociceptors. This approach is likely to prove useful in identifying other novel pronociceptive mediators in IBD and other inflammatory diseases.

## Conflict of interest statement

Dr Paul Wright is an employee of LifeArc. Dr David Bulmer has received research funding from LifeArc.

## Appendix A. Supplemental digital content

Supplemental digital content associated with this article can be found online at http://links.lww.com/PAIN/B984, http://links.lww.com/PAIN/B993, http://links.lww.com/PAIN/B994, http://links.lww.com/PAIN/B995 and http://links.lww.com/PAIN/B996.

## Supplementary Material

SUPPLEMENTARY MATERIAL
