# Supplementary material for: Transcriptomic profiling reveals a pronociceptive role for angiotensin II in inflammatory bowel disease
Source: Pain. 2024 Jan 29;165(7):1592–604. doi: 10.1097/j.pain.0000000000003159 (PMC11190897; doi:10.1097/j.pain.0000000000003159)
Supplement: SUPPLEMENTARY MATERIAL [file jop-165-1592-s004.docx]

**Supplemental table 1: Metadata for each patient group.**

**Supplemental table 2: List of prescribed treatments for each patient.** NTR: no treatment.
